# Treatment Delays for Patients With Acute Ischemic Stroke in a Rural Arkansas Emergency Department: A Retrospective Chart Review

**DOI:** 10.7759/cureus.61103

**Published:** 2024-05-26

**Authors:** Ahmed Muhsin, Brent Blackburn

**Affiliations:** 1 Research, Oceania University of Medicine, Texas, USA; 2 Medical School, Oceania University of Medicine, Apia, WSM

**Keywords:** teleneurologist, door to needle, treatment times, iv r-tpa, ischemic stroke

## Abstract

Objective

The goal of this study is to evaluate treatment times regarding acute ischemic stroke and identify barriers to implementing stroke care at the Ouachita County Medical Center Emergency Department.

Methods

A retrospective review of medical records was conducted of patients who presented with acute ischemic stroke to the Ouachita County Medical Center Emergency Department between 2020 and 2023 and received intravenous (IV) r-tPA. The primary focus of this study was to analyze door-to-needle time with IV r-tPA. To determine areas of improvement, this study examined door-to-initial physician evaluation, door-to-CT, door-to-tele neurologist evaluation, and door-to-IV r-tPA administration.

Results

A total of 26 patients who received treatment with IV r-tPA for acute ischemic stroke were included in this study. Twenty-three patients (88%; n=26) received IV- r-tPA within the recommended 60-minute window with a mean treatment time of 44.5 minutes. The mean door-to-physician evaluation time for patients presenting with acute ischemic stroke was 1.81 minutes. All patients received CT scans within 28 minutes of arrival with the mean time being 5.08 minutes. Teleneurologist evaluation was initiated within 59 minutes of presentation with a mean time of 25.19 minutes.

Conclusion

Evaluation of treatment times at the Ouachita County Medical Center Emergency Department confirms that stroke care received at this facility adheres to the recommendations outlined by the American Stroke Association. Nevertheless, clinicians should always strive for improvement. Through extensive evaluation of the treatment process, we were able to provide recommendations to further decrease treatment times and improve overall clinical outcomes.

## Introduction

Background

In the United States, stroke is a leading cause of long-term disability and the fifth leading cause of death, affecting nearly 795,000 annually each year [1]. On average, someone in the United States has a stroke every 40 seconds, resulting in 411 stroke-related deaths each day [2]. Globally, 15 million people suffer from a stroke each year. Of these, five million die and an additional five million suffer permanent disability [3]. These numbers are expected to steadily rise with a 34% increased incidence of stroke in the coming decades [4].

Patients who are fortunate enough to survive an event are faced with significant challenges not only from a rehabilitation standpoint but also from the financial impact associated with this disease. Stroke-related costs in the United States came to nearly $56.5 billion between 2018 and 2019 [5]. This estimate includes not only the cost of health care services but also the loss of income associated with disability. Because of the significant prevalence and financial impact of the disease, researchers have worked diligently to identify more effective means to combat this epidemic.

Importance

A stroke, or cerebrovascular accident, is a neurological condition characterized by an acute compromise of the cerebral perfusion or vasculature [6]. This compromise can be classified as either ischemic or hemorrhagic, with approximately 87% being classified as ischemic [7]. During an ischemic stroke, the affected vessel becomes occluded due to a thrombotic or embolic event [6]. Following this occlusion, tissue distal to the site is deprived of the oxygen needed for normal cellular function. Affected cells cease neuronal function within seconds of ischemia and undergo irreversible necrosis within five minutes [8].

Following an ischemic stroke, two areas of ischemia develop, the core and the penumbra [9]. The core receives nearly 10-25% of normal blood flow from the affected vessels resulting in irreversible necrosis. The penumbra, which is ischemic tissue surrounding the core, receives blood from collateral circulation, delaying the completion of the infarct [10]. These neurons are considered salvable if reperfused within a timely manner and are the target of treatment [11].

Alteplase (a recombinant tissue plasminogen activator (tPA), thrombolytic agent, and the only FDA-approved pharmacologic treatment for acute ischemic stroke) can lyse the offending clot and restore blood flow to ischemic areas preventing further neuronal loss if administered promptly [12]. The American Heart Association recommends the administration of Alteplase (tPA) within 4.5 hours of symptom onset in qualifying patients [13]. Patients who receive this therapy within the recommended time frame are 1.9 times more likely to have a favorable outcome with earlier treatment being directly correlated with improved clinical outcomes [13]. This has led the American Heart Association to adopt a recommended door-to-needle time of < 60 minutes for the administration of intravenous (IV) r-tPA [13]. Due to the significance of the research supporting timely intervention, it is imperative that hospitals evaluate current treatment times and identify any barriers that may result in prolonged treatment.

Goals of this study

The goal of this study is to evaluate emergency department (ED) treatment times regarding acute ischemic stroke and identify barriers to implementing stroke care. The primary focus of this study will be to analyze door-to-needle time with intravenous r-tPA for patients who present with acute ischemic stroke and meet the criteria for intervention. To determine areas of improvement, this study will assess the following areas of the treatment process: door-to-physician evaluation, door-to-CT, door-to-tele neurologist evaluation, and door-to-IV r-tPA administration.

## Materials and methods

Study design

A retrospective review of medical records was conducted of patients with acute ischemic stroke who presented to the ED of Ouachita County Medical Center and received IV r-tPA between 2020 and 2023. The study objectives were explained, and approval was granted by the OCMC administration and Oceania University of Medicine institutional review board (OUMHREC24.023). Because this study represented a retrospective review of medical records, the informed consent requirement was waived by hospital officials and the university. Data extraction and interpretation were conducted in a manner to ensure patient confidentiality in compliance with the HIPPA Privacy Rule [14].

Setting

This study was conducted at Ouachita County Medical Center (OCMC) in Camden, AR, a rural community residing in Ouachita County. The hospital serves as the sole medical facility of Ouachita County and serves the 23,382 citizens residing within the county as well as those in the surrounding communities. OCMC is an 82-bed short-term acute care hospital with a 10-bed level-IV trauma center ED. The ED has 24-hour in-house physician coverage as well as nursing, radiology, and laboratory supporting staff. Annual ED visits were 12,000 in 2023, representing a 5% increase from the year prior [15].

Selection of participants

Patients who presented to the ED between 2020 and 2023 with a diagnosis of acute ischemic stroke were included for analysis according to the following inclusion criteria: diagnosis of acute ischemic stroke confirmed by an ED physician/teleneurologist, non-contrast CT of head ruling out other etiologies (hemorrhage, mass, etc.), and a deemed candidate for IV r-tPA administration as identified by the American Heart Association’s criteria for treatment [11]. Those patients who failed to meet the criteria for treatment of acute ischemic stroke with IV r-tPA (last known well time >4.5 hours, coagulopathy, uncontrolled HTN, active bleeding, etc.) were excluded from this study. Demographics for patients included in this study are listed in Table 1.

**Table 1:** Demographics EMS: Emergency medical services; POV: privately owned vehicle

Variable	Age/Frequency (%)
Age (N=26)	
Mean	63.9 Years
Median	60 Years
Mode	54 & 75 Years
Range	38-94 Years
Sex (N=26)	
Male	11 (42%)
Female	15 (58%)
Ethnicity (N=26)	
African American	9 (34%)
Caucasian	17 (66%)
Mode of Arrival (N=26)	
EMS	11 (42%)
POV	15 (58%)
Comorbidities (N=26)	
Hypertension	22 (85%
Hyperlipidemia	6 (23%)
Coronary Artery Disease	9 (35%)
Tobacco Use	8 (31%)

Data collection

A review of the electronic medical records was executed for all acute ischemic stroke patients who presented within the four-year timeframe and were treated with IV r-tPA. To ensure the suitability of each candidate selected for the study, a detailed chart review was conducted for each selected patient by the emergency department medical director and the hospital stroke team coordinator.

Outcome measures

The primary outcome of this study is to evaluate ED performance regarding stroke treatment times and identify barriers that result in delayed treatment. Secondary outcomes measured include time of arrival (AM vs PM) (Weekday vs Weekend) to determine if treatment times are consistent between each group.

Data analysis

Statistical analysis was conducted using Statisty statistical software [16]. Descriptive statistics for data are reported as mean with SD, 95% confidence interval of mean, median, and mode. Identified variables are also reported as percentages and statistical ranges. In order to test the null hypothesis and identify differences between the groups with regard to arrival time (AM vs PM, weekday vs weekend), a t-test was conducted. 

## Results

During this study (January 1st, 2020 - December 31st, 2023), a total of 26 patients received IV r-tPA for the treatment of acute ischemic stroke. Of these 26 patients, six presented to the ED between 7:00 PM and 7:00 AM (23%) while 10 presented on Saturday or Sunday (38.5%). Of the 26 patients who received IV r-tPA, 23 (88%) received treatment before the target door to treatment time of < 60 minutes.

**Figure 1:**
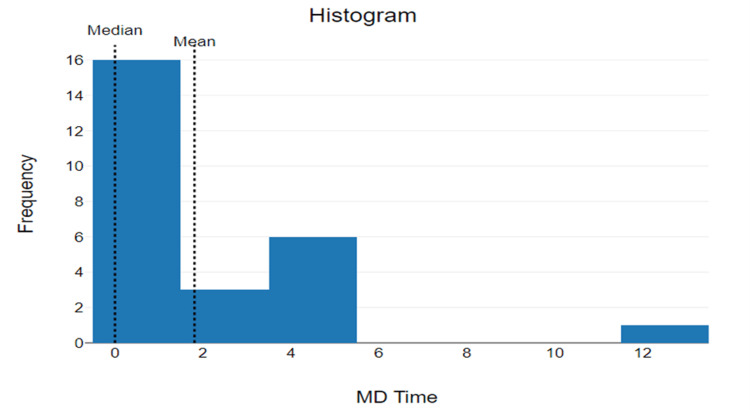
Door-to-physician The x-axis reflects time (minutes) elapsed from arrival to initial physician evaluation. The y-axis reflects the frequency (number of patients) who were evaluated within the corresponding time frame.

The median door-to-physician time for patients with suspected acute ischemic stroke who received IV r-tPA was 0 minutes with the mode also being 0 minutes (N=16) (44%). The statistical range of door-to-physician times was 12 (12-0) (Figure 1)*.* The mean door-to-physician time was 1.81 minutes with a standard deviation of 2.83 and 95% CI of mean 0.72 to 2.9 minutes.

**Figure 2:**
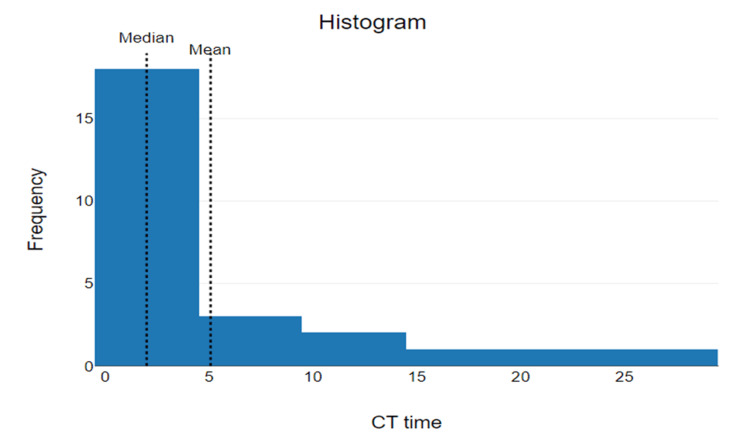
Door-to-CT The x-axis reflects time elapsed (minutes) from arrival to CT. The y-axis reflects the frequency (number of patients) who received CT within the corresponding timeframe.

The median door-to-CT time for patients with suspected acute ischemic stroke who received IV r-tPA was 2 minutes with the mode being 0 minutes (n=9) (34.6%). The statistical range of door-to-CT time was 28 (28-0) (Figure 2). The mean door-to-CT time was 5.08 minutes with a standard deviation of 7.39 and 95% CI of mean 2.24 to 7.92 minutes.

**Figure 3:**
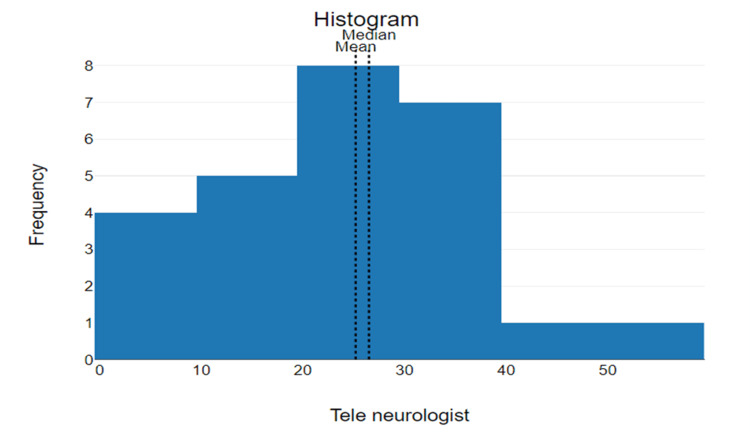
Door-to-teleneurologist The x-axis represents the time elapsed (minutes) from arrival to teleneurologist evaluation. The y-axis represents the frequency (number of patients) who received teleneurologist evaluation within the corresponding timeframe.

The median door-to-teleneurologist time for patients receiving IV r-tPA was 26.5 minutes with the mode being 17 minutes (N=2) (7.7%). The statistical range of door-to-teleneurologist was 57 (59-2) (Figure 3). The mean door-to-teleneurologist time was 25.19 minutes with a standard deviation of 13.58 and 95% CI of mean 19.91 to 30.41 minutes.

**Figure 4:**
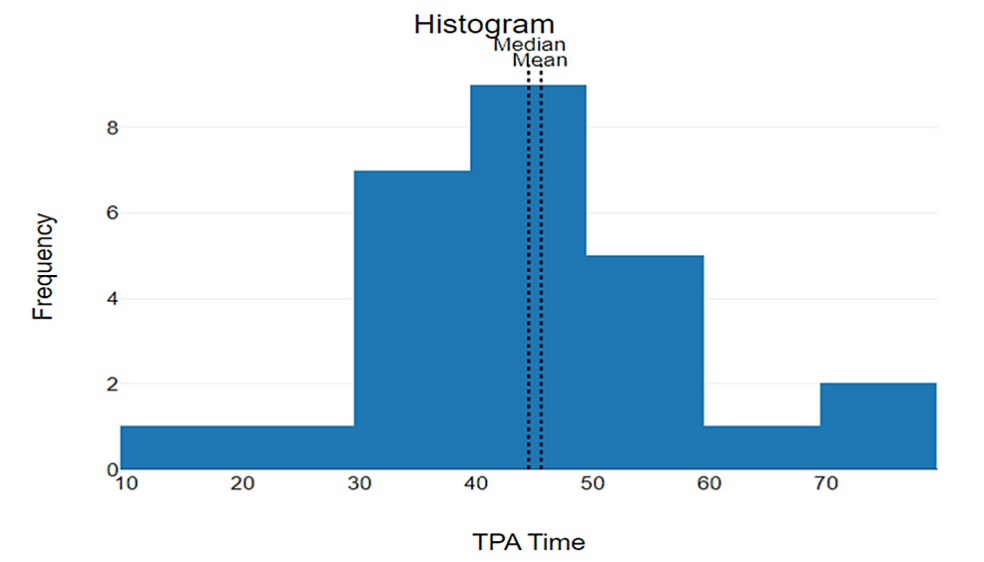
Door-to-needle (IV r-tPA) The x-axis represents the time elapsed (minutes) from arrival to administration of IV r-tPA. The y-axis represents the frequency (number of patients) who received treatment within the corresponding timeframe. tPA: Tissue plasminogen activator

The median door-to-IV r-tPA time was 44.5 minutes with the mode being 38 minutes (n=2) (7.7%). The statistical range of door-to-IV r-tPA was 58 (75-17) (Figure 4). The mean door-to-IV r-tPA time was 45.58 minutes with a standard deviation of 13.34 and 95% CI of mean 40.45 to 50.7 minutes.

Various mean treatment times were collected and analyzed based on the patient’s time of arrival (AM vs PM) (Weekend vs Weekday) (Table 2 and Table 3).

**Table 2:** Arrival time (AM vs PM) tPA: Tissue plasminogen activator

Interval	AM (N=20)	PM (N=6)	p-value (Two-Tailed)
Door-to-physician Mean (SD)	1.45 (3.05)	2 (1.91)	0.68
Door-to-CT Mean (SD)	4.5 (7.75)	5.71 (6.42)	0.73
Door-to-teleneurologist Mean (SD)	23.55 (12.96)	26.8 (16.74)	0.62
Door-to-IV r-tPA Mean (SD)	43.6 (13.02)	55.43 (14.91)	0.62

**Table 3:** Arrival time (weekday vs weekend) tPA: Tissue plasminogen activator

Interval	Weekday (N=16)	Weekend (N=10)	p-value (Two-Tailed)
Door-to-physician Mean (SD)	2.06 (3.34)	1.51 (1.96)	0.64
Door-to-CT Mean (SD)	5.75 (8.38)	3.7 (5.83)	0.51
Door-to-tele neurologist Mean (SD)	26.69 (14.68)	26.8 (16.74)	0.99
Door-to-IV r-tPA Mean (SD)	47.38 (14.78)	44.5 (11.35)	0.60

## Discussion

It is well established that an improved clinical outcome is time-dependent on successful recanalization with IV r-tPA [17]. Individuals who receive timely treatment have a 32 % relative increase in the likelihood of minimal or no disability at 90 days [18]. Because timely intervention is of the utmost importance, the American Heart Association has introduced recommendations regarding various aspects of the treatment process. These recommendations include door-to-physician evaluation of < 10 minutes, door-to-CT/MRI initiation of < 25 minutes, and door-to-needle of < 60 minutes [19]. All of these were met based on the mean times of this study. However, to fully comprehend the significance of these results, a detailed comparison with previous studies may provide additional insight.

Evaluation of the door-to-physician time in this study revealed a mean of 1.81 minutes and a median of 0 minutes. A 40-patient study conducted in 2012 found similar results with a median door-to-physician time of 5 minutes and a mean time of <10 minutes. However, this study revealed that only 7.5% of patients received IV r-tPA within the recommended timeframe of 60 minutes [20]. This raises a concern about the significance of timely physician evaluation regarding IV r-tPA administration times.

The Ouachita County Medical Center’s median door-to-CT time was 2 minutes with a mean of 0 minutes. A similar study published in 2022 evaluated door-to-CT time in 310 patients with ischemic stroke who presented to a central referral hospital in Indonesia and found a median door-to-CT time of 19.5 minutes [21]. The study did not evaluate door-to-needle times, so we were unable to determine if prolonged CT times had an effect on the IV r-tPA administration time.

Because telemedicine consultation is relatively uncommon in the academic setting, there were few studies available for comparison. A 2012 study revealed a mean door-to-teleneurologist time of 52.65 minutes with a median time of 51 minutes compared to the mean time of 25.19 minutes and 26.5 minutes median found at OCMC [20]. The study also found that only 7.5% of patients received IV r-tPA within 60 minutes upon arrival. This suggests that the door-to-teleneurologist time may have the largest effect on door-to-needle times.

Analysis of arrival time in regard to AM vs PM and weekday vs weekend revealed p values > 0.10 (Table 2 and Table 3*)*. This confirms that there is no significant difference between the groups, confirming the null hypothesis. 

Based on this study's findings, 88% (n=23) of patients treated at Ouachita County Medical Center had a door-to-needle time of < 60 minutes. In comparison, a study conducted in 2011 comprising a total of 25,000 patients found that less than one-third of patients receive treatment within the 60-minute timeframe [22]. A separate study conducted in Iran found the median door-to-needle time for patients treated with IV r-tPA to be 69 minutes compared to the median door-to-needle time of 44.5 minutes that was found in this study [23].

In 2020, the American Heart Association introduced the Stroke Honor Roll program which recognizes hospitals based on their cumulative door-to-needle times [24]. These levels are as follows: 

Target: Stroke Honor Roll: Door-to-needle times within 60 minutes for at least 75 percent of applicable patients.

Target: Stroke Honor Roll-Elite: Door-to-needle times within 60 minutes for at least 85 percent of applicable patients.

Target: Stroke Honor Roll-Elite Plus: Door-to-needle times within 45 minutes for at least 75 percent of applicable patients and door-to-needle times within 30 minutes for at least 50 percent of applicable patients.

In addition, the American Heart Association has implemented Stroke Phase III which recommends hospitals achieve a door-to-needle time of < 60 minutes for at least 85% of applicable patients [25]. By achieving a door-to-needle time of < 60 minutes in 88% of applicable patients, the Ouachita County Medical Center Emergency Department adheres to the recommendations of Stroke Phase III and meets criteria for Stroke Honor Roll-Elite recognition. 

Limitations

Although the findings of this study outline key elements in overall stroke care, they are not without limitations. The largest and perhaps the most significant limitation of the study is that the data was retrospectively collected from the electronic medical record. Therefore, the validity of these results relies on the assumption that documentation is thorough and accurate.

Another limitation of the study is that we cannot account for variables that are out of the control of medical staff and may delay the treatment process. These variables are often case-specific and would be virtually impossible to incorporate into the study. A few examples include delays in determining the last known well time, delays in obtaining past medical history (including home medications), other critical patients in the ED, delays in CT reports, technical difficulties during telemedicine consultations, etc. Ultimately, when analyzing these results, we must keep in mind that any radical outlier regarding treatment times may be the result of uncontrollable variables that cannot be captured through the electronic medical record.

Lastly, because the sample size of patients receiving IV r-tPA is relatively small (n=26), the mean times can be significantly altered by a small number of cases. Out of the 26 cases we reviewed, there were two (74 and 75 minutes) in which the door-to-IV r-tPA administration time was significantly greater than the remaining cases. Because of the smaller sample size, these outliers were able to have a greater effect on the overall mean treatment time and may have been the result of one of the previously listed uncontrollable variables.

Recommendations

To investigate further into various aspects that may delay treatment times, the researcher recommends that a standardized stroke care template be incorporated into the EMR. This template should include door-to-physician time, door-to-CT time, door-to-CT report time, door-to-teleneurologist consult initiated time, door-to-teleneurologist evaluation time, door-to-treatment decision time, door-to IV r-tPA administration time, and a miscellaneous section to identify any patient-specific barriers to treatment. By capturing this additional data, administrators may be able to identify specific barriers to overall door-to-needle times and make appropriate recommendations.

## Conclusions

Overall, the findings of this study reveal that patients presenting with acute ischemic stroke to the OCMC ED are receiving a standard of care that is consistent with the American Heart Association's recommendations. However, as with all aspects of medicine, there is always room for improvement. Future research should be conducted to complete a more in-depth analysis of treatment times to identify specific areas of improvement. Through future research and appropriate intervention, the Ouachita County Medical Center Emergency Department could reach Stroke Honor Roll-Elite Plus status as outlined by the American Heart Association and ultimately provide a higher level of care to patients presenting with acute ischemic stroke.
